# Spin‐Polarized Metallic Metal−Organic Framework With High Conductivity and Half‐Metallic Behavior

**DOI:** 10.1002/advs.78726

**Published:** 2026-11-09

**Authors:** Saqib Kamal, Kuang‐Hsin Hsu, Arif I. Inamdar, Hemanth Kumar Bangolla, Chi‐Jia Lin, Erdembayalag Batsaikhan, Tzuoo‐Tsair Luo, Ming‐Hsi Chiang, Yen‐Hsiang Liu, Ruei‐San Chen, Batjargal Sainbileg, Michitoshi Hayashi, Chen‐Hsiung Hung, Kuang‐Lieh Lu

**Affiliations:** ^1^ Institute of Chemistry Academia Sinica Taipei Taiwan; ^2^ Department of Chemistry Fu Jen Catholic University New Taipei Taiwan; ^3^ Department of Chemistry Emerson University Multan Multan Pakistan; ^4^ Graduate Institute of Applied Science and Technology National Taiwan University of Science and Technology Taipei Taiwan; ^5^ Center For Condensed Matter Sciences National Taiwan University Taipei Taiwan; ^6^ Center of Atomic Initiative For New Materials National Taiwan University Taipei Taiwan; ^7^ National Center for Theoretical Sciences Taipei Taiwan

**Keywords:** conductivity, electronic structure, half‐metal, metallic conductivity, metal‐organic framework, periodic density functional theory, photoconductivity, single crystal, spin polarization

## Abstract

The establishment of new concepts for next‐generation conductive materials remains both highly desirable and scientifically challenging. Herein, we report a Cu‐based metal–organic framework (MOF), ([Cu(6Hmna)]_n_ (**1**, 6Hmna = 6‐mercaptonicotinic acid)), featuring metal–sulfur chains that exhibit intrinsic metallic behavior. Compound **1** exhibits a high electrical conductivity of up to 120 S cm−1, indicating a high electrical conductivity for a MOF in its crystalline form. The metallic transport characteristics are confirmed by temperature‐dependent conductivity measurements. In addition, this Cu‐MOF demonstrates highly efficient photoconductivity, with an outstanding photocurrent responsivity reaching 8100 A W^−1^ under infrared light illumination. Our theoretical DFT calculations predict that compound **1** exhibits an exotic spin‐polarized electronic structure, in which one spin channel is metallic while the other remains semiconducting. This unique electronic configuration is particularly attractive for applications in spintronics and quantum technologies, as well as in optoelectronic and photoelectrochemical devices.

## Graphical abstract

By establishing a progression from insulating to semiconducting and ultimately to metallic MOFs, this study not only introduces a new design paradigm based on quantum spin‐polarized electronic structures, but also represents a significant breakthrough toward the realization of next‐generation conductive materials.

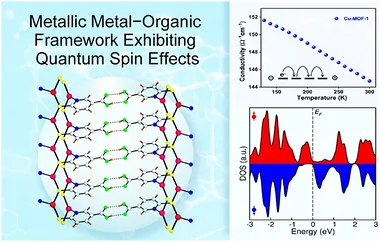

## 1 Introduction

Electrically conductive metal–organic frameworks (cMOFs) have attracted considerable attention in materials science owing to their diverse functional potential, including applications in chemical sensing, electrocatalysis, energy storage, and emerging roles in microelectronic and optoelectronic devices [1, 2, 3, 4, 5]. Efficient charge transport in metal–organic frameworks (MOFs) relies on the establishment of well‐defined conductive pathways that ultimately govern the macroscopic electrical conductivity of these materials. Although the vast majority of MOFs behave as electrical insulators owing to poor orbital overlap between redox‐inactive metal centers and ligand π‐systems, various charge‐transport mechanisms have been identified in conductive MOFs, including through‐bond conduction along metal–ligand coordination bonds, through‐space interactions (such as π–π stacking and other weak supramolecular forces), and extended π‐conjugation across the framework. These mechanisms collectively enable electron delocalization throughout the crystalline lattice. Organic linkers play a pivotal role by supplying extended π‐systems and soft donor atoms (e.g., nitrogen‐ or sulfur‐functionalized ligands), which facilitate strong d–π orbital overlap and efficient charge delocalization. Complementarily, redox‐active metal nodes provide appropriate d‐orbital energies for hybridization, promote mixed‐valence states, and dictate interlayer stacking arrangements that influence the charge transport. This design paradigm, extensively discussed in foundational reviews and structural studies, has elevated MOF conductivities by several orders of magnitude, positioning conductive MOFs as promising platforms for applications in electrocatalysis, energy storage, and electronic devices [6, 7, 8, 9, 10, 11].

Semiconducting MOFs have been successfully developed, opening new avenues for both fundamental investigation and practical applications [12, 13, 14, 15]. In particular, these materials have attracted considerable interest owing to their intrinsic electronic properties, including high charge‐carrier mobility, electrical conductivity, and photoconductivity [16, 17]. Among these characteristics, electrical conductivity is especially compelling and has emerged as a central focus of advanced MOF research. MOFs with enhanced conductivity are promising candidates for a broad range of electronic applications, including memory devices, electrically transduced sensors such as impedance‐ and capacitance‐based sensors, and energy storage systems [18, 19, 20, 21]. Despite impressive progress in improving the electrical conductivity of MOFs, a true transition from semiconducting to metallic behavior remains unrealized, highlighting a critical conceptual and materials challenge in the field.

Spin is a fundamental quantum property of electrons, providing an additional degree of freedom that can profoundly influence electronic properties, including electrical conductivity [22, 23, 24, 25]. In transition‐metal systems, electron transport is intrinsically governed by spin‐dependent electronic states, particularly in 3d metals such as copper [26, 27]. In the context of MOFs, exploiting spin‐driven design strategies offers a promising, largely unexplored route to enhanced electrical conductivity and emergent quantum functionalities [28, 29, 30].

Although MOFs have been extensively investigated as semiconductors, their potential as metallic or half‐metallic materials remains largely untapped. Half‐metallic materials, characterized by spin‐polarized electronic structures in which one spin channel is metallic while the other is semiconducting, are of exceptional interest for spintronics and quantum technologies [31, 32, 33]. However, realizing such spin‐polarized electronic behavior in MOFs has remained a formidable challenge, owing to their predominantly insulating electronic structures.

In this study, we demonstrate that a self‐assembled Cu‐based MOF featuring (–Cu–S–)_n_ chains exhibits metallic behavior, as established by a combination of experimental measurements and theoretical analyses. Quantum‐mechanical simulations further reveal that this Cu‐MOF possesses an exotic half‐metallic electronic structure, exhibiting metallic or semiconducting behavior depending on the spin channel. Notably, half‐metallicity is exceedingly rare in MOF materials and is highly sought after for emerging applications in optoelectronics, spintronics, and quantum technologies. Motivated by theoretical predictions, experimental investigations of single crystals confirm that this Cu‐MOF exhibits intrinsic metallic behavior and high electrical conductivity. Collectively, these findings broaden the functional landscape of metal–organic frameworks beyond conventional semiconducting regimes and provide a robust design strategy for creating metallic inorganic–organic hybrid materials with precisely tunable electronic properties.

## 2 Experimental Section

### 2.1 Materials and Instruments

The chemical reagents used for carrying out these studies were purchased commercially and were used without any further purification. Diffraction measurements for compound **1** were performed using a Brucker‐Nonius Kappa CCD diffractometer with graphite‐monochromated Mo Kα radiation (λ = 0.7107 Å). The crystallographic data collection parameters for compound **1** are summarized in Table S1 (ESI) and have been deposited in the Cambridge Structural Database under CCDC number 2487608. The structure was solved by direct methods and refined by full‐matrix least‐squares on *F^2^* using the SHELXL‐97 program. All non‐hydrogen atoms were refined anisotropically, while the hydrogen atoms were placed in ideal and calculated positions, with isotropic thermal parameters riding on their respective carbon atoms. Infrared spectra were performed in the solid state (KBr pellets) using a Perkin‐Elmer PARAGON 1000 FT‐IR spectrometer ranging between 4000–400 cm–^1^. CHN elemental analysis was conducted on a Perkin‐Elmer 2400 elemental analyzer. Thermogravimetric (TG) analyses were done under nitrogen with a heating rate of 10°C min–^1^ on a Perkin‐Elmer TGA‐7 TG analyzer. Powder diffraction studies were recorded using a Siemens D‐5000 diffractometer at 40 kV, 30 mA for Cu Kα (λ = 1.5406 Å), with a step size of 0.015 and a scan speed of 0.15 s per step.

### 2.2 Fabrication and Characterization of Single‐Crystal Devices

Single‐crystal devices were fabricated via the mechanical exfoliation method using dicing tape. The exfoliated crystals were transferred onto SiO_2_ (300 nm)/n^+^‐Si substrates pre‐patterned with Ti/Au electrodes. To ensure ohmic electrical contact, the platinum (Pt) electrodes were deposited onto selected high‐quality crystals using a focused‐ion beam (FIB‐FEI Quanta 3D FEG) technique. The voltage and current were maintained at 30 kV and 100 pA, respectively, for ion beam decomposition of the Pt precursor. The device dimensions were determined from field‐emission scanning electron microscopy (FESEM; Hitachi S3000H) images. The thickness of the single crystals was estimated from atomic force microscopy (AFM; Bruker Dimension Icon) height‐profile measurements. The electrical characterization in the temperature range 100−300 K was carried out in an ultralow current leakage cryogenic probe station (LakeShore Cryotronics TTP4). A semiconductor system (Keithley 4200‐SCS) was employed as the source–measurement unit. To perform the photoconductivity measurements, a diode laser (λ = 808 nm) was used as an excitation light source. To broaden the laser beam to ∼25 mm^2^ and minimize errors in the calculated light intensity, a UV holographic diffuser was used. The calibrated power meter (Ophir Nova II) with a silicon photodiode head (Ophir PD300‐UV) was used for incident laser power measurements.

### 2.3 Synthesis and Characterization

A mixture of CuCl_2_·H_2_O (17.0 mg, 0.1 mmol), 6‐mercaptonicotinic acid (15.0 mg, 0.1 mmol), dimethylformamide (5 mL), and CH_3_CN (3 mL) was sealed in a Teflon‐lined stainless steel Parr acid digestion bomb and heated at 160°C for 72 h and then allowed to slowly cool to room temperature at a rate of 6 h/°C. Orange rectangular‐shaped crystals of compound **1** were obtained in a 48.5% yield. The solid product was isolated on a filter, washed with water, and dried at ambient temperature.

Elementary anal. Calcd (%) for: C, 33.10; H, 1.85; N, 6.43; S, 14.73. Found (%): C, 32.99; H, 1.87; N, 6.58; S, 14.50. IR data (KBr, cm–^1^): 3681(w), 2810 (w), 2553 (s), 1682 (s), 1424 (s), 1189(s), 1916(m), 835(w), 751(s), 639 (w), 560 (s), 481(s).

### 2.4 Computational Methods

First‐principles calculations based on periodic density functional theory (DFT) were performed using the Vienna Ab‐Initio Simulation Package (VASP 5.4.4) with the projector‐augmented wave (PAW) potentials [34, 35]. Spin‐polarized Perdew‐Burke‐Ernzerhof calculations with an on‐site Hubbard correction (PBE+U, U = 4 eV), together with Grimme's D3 dispersion correction for van der Waals (vdW) interactions, were employed as the exchange‐correlation functional throughout. A plane‐wave cut‐off energy of 520 eV and a 14 × 8 × 2 Monkhorst‐Pack k‐point mesh were used for Brillouin‐zone sampling. The Cu‐MOF crystal structure obtained from experimental data was fully relaxed until the total energy and atomic forces converged to be 0.1 µeV and 1 meV/Å, respectively.

## 3 Results and Discussion

### 3.1 Synthesis and Crystal Structure of Cu‐Based MOF 1

In this study, compound [Cu(6Hmna)]_n_ (**1**) was synthesized by reacting CuCl_2_·H_2_O with 6‐mercaptopyridine‐3‐carboxylic acid (6Hmna) at 160°C for 3 days in a DMF–CH_3_CN solvent system under hydrothermal conditions (Scheme 1). The rational selection of organic ligands bearing specific coordinating functionalities is critical for achieving unique framework architectures that enable continuous charge‐transport pathways throughout the crystal lattice. The 6Hmna ligand was chosen because it incorporates both thiol (–SH) and carboxylic acid (–COOH) groups, which promote the formation of one‐dimensional (–M–S–)_n_ chains that stack along the *c*‐axis. These chains facilitate charge transport through strong orbital overlap between sulfur p‐orbitals and metal d‐orbitals. In addition, the carboxylate moiety provides versatile coordination modes that contribute to the formation of a structurally robust MOF.

**SCHEME 1:**
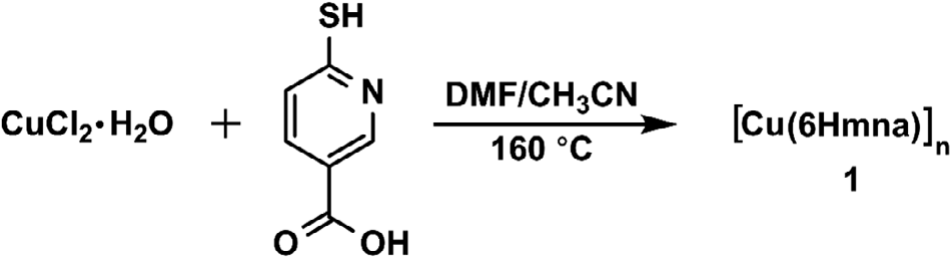
Synthesis of compound **1**, (6Hmna denotes 6‐mercaptonicotinic acid).

Single‐crystal X‐ray diffraction analysis revealed that compound **1** crystallizes in the monoclinic space group P2_1_/c (Table S1, ESI). The asymmetric unit (Figure 1a) comprises a Cu(I) center coordinated by two sulfur atoms (S1A, S1B) from two independent 6Hmna ligands and one nitrogen atom (N1’) from a third 6Hmna ligand, forming a {CuS_2_N} trigonal‐planar unit. Charge neutrality is achieved through deprotonation of the thiol (–SH) group of the 6Hmna ligand upon coordination to the Cu(I) center. As illustrated in Figure 1b, these {CuS_2_N} units are linked through thiolate bridges to generate one‐dimensional (–Cu–S–)_n_ chains with alternating Cu and S atoms arranged in a zigzag configuration along the *a* axis (Figure 1c). Hydrogen‐bonding interactions between neighboring carboxylic acid groups further help stabilize the framework. Each 6Hmna ligand adopts a µ_3_‐κN:κS:κS coordination mode, coordinating one Cu(I) ion via its pyridyl nitrogen (Cu–N = 1.991 Å) and simultaneously bridging two adjacent Cu(I) centers through its deprotonated thiolate sulfur (Cu–S = 2.194 and 2.292 Å). Within the thiolate‐bridged Cu(I) chain, the Cu···Cu separations are 3.893 Å, while the interchain Cu···Cu distances are as short as 3.064 Å. The three‐dimensional packing structure (Figure 1d and Figure S1, ESI) highlights the interchain stacking, short Cu···Cu contacts, and hydrogen‐bonding interactions between neighboring chains.

**FIGURE 1:**
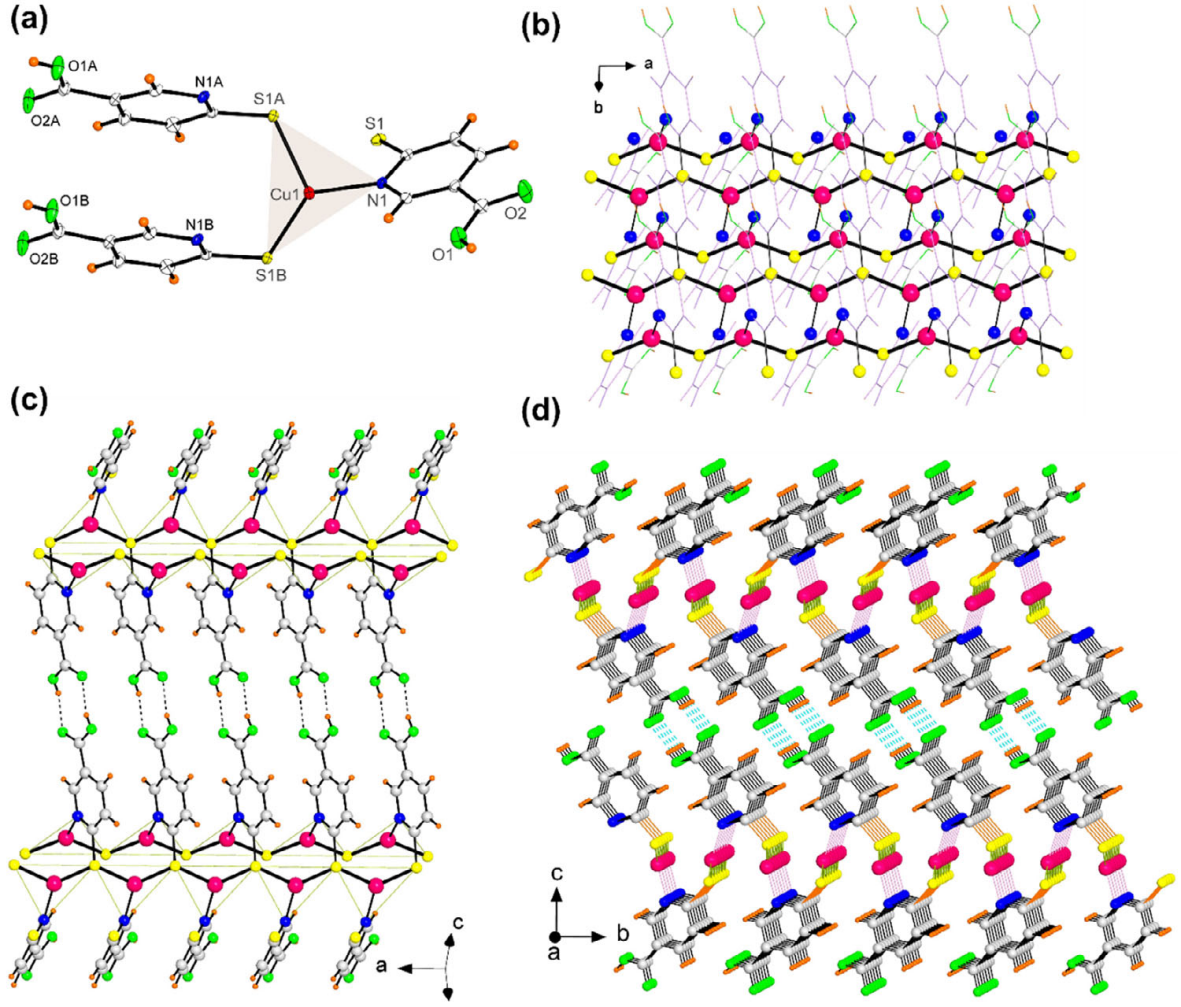
(a) Asymmetric unit of **1**, with atom color code: Cu (pink), S (yellow), N (blue), and O (green). (b) View of (–Cu–S–)_n_ chains projected onto the *ab* plane along the *c*‐direction, showing chain propagation along the *a*‐axis. (c) Perspective view of the (–Cu–S–)_n_ chains, highlighting the Cu–S connectivity and interchain arrangement. (d) Three‐dimensional packing diagram viewed along the *a*‐axis, illustrating interchain stacking and hydrogen‐bonding interactions (water‐blue) between neighboring chains.

### 3.2 Powder X‐ray Diffraction and Thermogravimetric Analysis

Powder X‐ray diffraction (PXRD) measurements were carried out to evaluate the phase purity of the compound. At room temperature, the experimental diffraction pattern closely matches the simulated pattern derived from single‐crystal data, confirming the formation of a phase‐pure material (Figure 2b). The framework exhibits high thermal stability, remaining structurally intact up to 340°C. This robustness can be attributed to the continuous Cu–S chains, the chelating coordination mode of the 6Hmna ligand, and π–π interactions between pyridyl groups, together with stabilizing hydrogen‐bonding interactions between neighboring carboxylic acid/carboxylate groups, which collectively reinforce the overall framework architecture (Figure 1).

**FIGURE 2:**
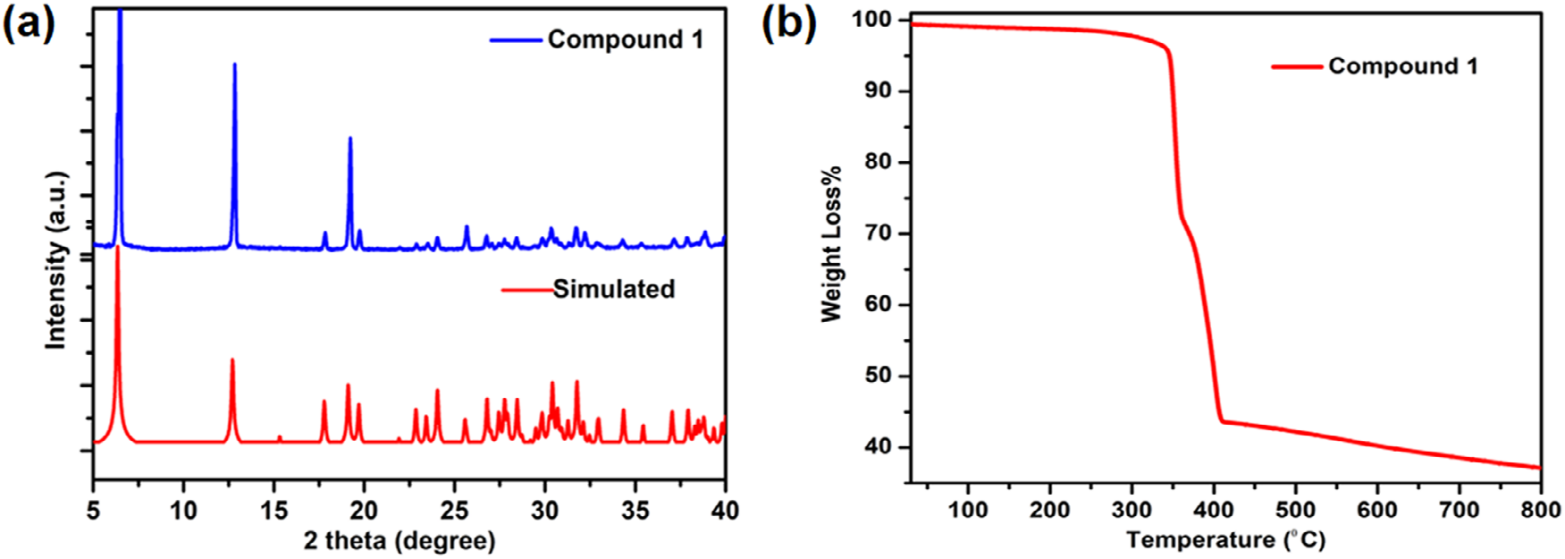
(a) Experimental powder X‐ray diffraction (PXRD) pattern (top) compared with the simulated pattern (bottom). (b) Thermogravimetric analysis (TGA) curve of compound **1**.

### 3.3 Electronic Transport Properties

The current–voltage (*I–V*) characteristics of a representative Cu‐MOF single crystal device with two‐terminal electrical contacts fabricated by focused ion beam (FIB) (device No. Cu‐MOF‐1), together with the corresponding AFM image (inset), are shown in Figure 3a. The observed linear *I–V* behavior indicates the formation of good ohmic contacts between the Cu‐MOF crystal and the Pt electrodes. The electrical conductivity (σ) was calculated using the equation: σ = *G*(1/*A*) = *G*(1/*wt*), where, *l* (1.1 ± 0.05 µm), *w* (1.1 ± 0.1 µm), and *t* (0.56 ± 0.01 µm) denote the length, width, and thickness of the conduction channel, respectively [36]. Here, *G* represents the electrical conductance obtained from the linear fit of the *I–V* curve. Based on these parameters, the conductivity of device Cu‐MOF‐1 was determined to be 120 ± 12 S cm^−1^, representing the high conductivity value for single‐crystalline MOF materials [12, 15, 16, 37−44]. Consistently high conductivity was also observed for a second device (device No. Cu‐MOF‐2), which exhibited a conductivity of 90 ± 10 S cm^−1^ with channel dimensions of *l* = 1.1 ± 0.05 µm, *w* = 1.6 ± 0.14 µm, and *t* = 0.2 ± 0.01 µm. The high conductivity is likely attributed to strong electronic coupling between the Cu(I) *d* orbitals and the sulfur p orbitals within the (–Cu–S–)_n_ chains, complemented by additional weak interactions, including π–π stacking and hydrogen‐bonding interactions [12].

**FIGURE 3:**
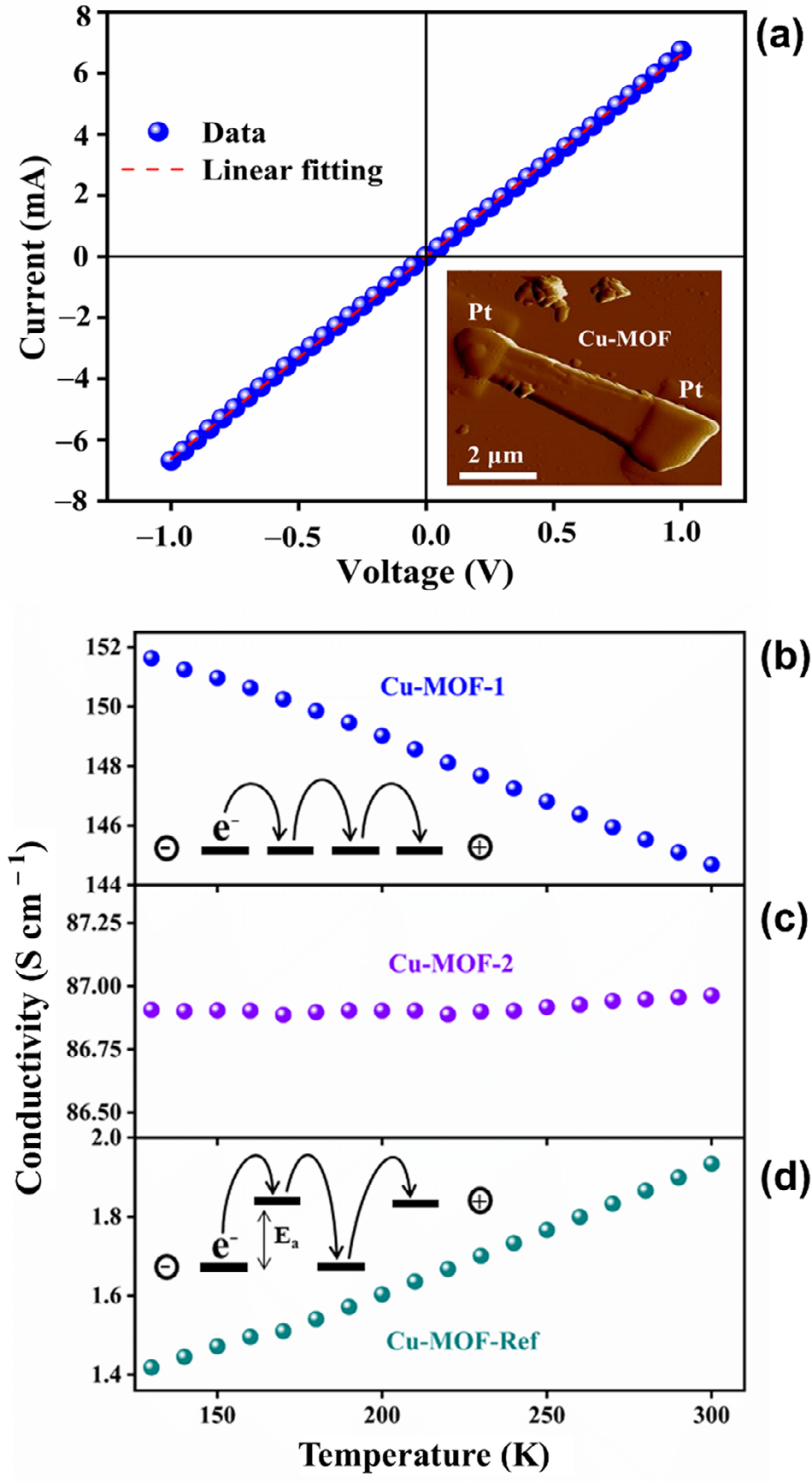
(a) Current–voltage (I–V) characteristics of a Cu‐MOF single‐crystal device (Cu‐MOF‐1). The inset shows an AFM image of the device fabricated by focused ion beam (FIB). Temperature‐dependent conductivity of (b) Cu‐MOF‐1, (c) Cu‐MOF‐2, and (d) a previously reported Cu‐MOF [12]. The insets in panels (b) and (d) schematically illustrate charge‐transport mechanisms: metallic transport without thermal activation and semiconducting transport requiring thermal activation energy (E_a_), respectively.

The temperature‐dependent electrical conductivity of the Cu‐MOF single crystals was investigated over the range of 130−300 K. For Cu‐MOF‐1 (Figure 3b), the conductivity increases monotonically with decreasing temperature, a hallmark of metallic transport behavior. In contrast, Cu‐MOF‐2 (Figure 3c) exhibits an almost temperature‐independent conductivity, suggesting that the activation energy of carriers is near zero. These observations of metallic transport and zero‐activation energy are distinct from previous reported semiconducting MOFs, and related metal–organic materials, which typically exhibit thermally activated transport with activation energies spanning a broad range of 1.6–293 meV (Table S2, ESI) [12, 14, 15, 16, 37, 44]. The emergence of metallic transport and near‐zero activation energy in the present system indicates that thermal energy is no longer required to activate charge carriers from bound states. This represents charge carriers in the MOF crystal that exhibit transport behavior analogous to that of free carriers in conventional metals. The presence of near‐zero bound states associated with the (–Cu–S–)_n_ chains extended along the *a*‐axis and planes stacked along the *c*‐axis is likely serve as the primary source of these free carriers. The metallic behavior is also consistent with the high conductivity observed in this Cu‐MOF, further corroborating its intrinsic metallic nature.

For comparison, a representative temperature‐dependent conductivity profile of a previously reported semiconducting Cu‐MOF, {[Cu_2_(6Hmna)(6mn)]•NH_4_}_n_, featuring (–Cu–S–)_n_ planes from our earlier study [12], is included in Figure 3d. In general, charge transport in MOFs can be described by through‐bond, through‐space, and through‐guest hopping mechanisms [12]. Accordingly, an activation energy (*E*_a_) is required to facilitate charge transfer from one bound state to another, as schematically illustrated in the inset of Figure 3d. In stark contrast, the metallic transport behavior observed for the present Cu‐MOF (Cu‐MOF‐1) indicates that charge hopping proceeds without the need to overcome an activation barrier, as depicted in the inset of Figure 3b. Under these circumstances, the electrical conductivity increases with decreasing temperature due to reduced charge‐carrier scattering and enhanced carrier mobility, a characteristic feature of metallic conduction.

For comparison, a structurally related Cu–S framework compound reported previously exhibited a much lower conductivity of 5 × 10^−3^ S cm^−1^ [20]. In contrast, our results show that the present Cu‐MOF displays an exotic metallic character, characterized by a conductivity of 120 ± 12 S cm^−1^ and an effectively zero activation energy. Nevertheless, the conductivity is lower than the reported metallic MOFs based on lanthanide, whose conductivity exceeds 1000 S cm^−1^ [45]. This metallic transport behavior is likely enabled by multiple complementary charge‐transport pathways, including direct through‐bond conduction along the Cu–S–Cu chains and through‐space transport mediated by weak interchain interactions, such as π–π stacking, hydrogen bonding, and short Cu···Cu contacts between neighboring Cu–S–Cu chains. Importantly, this unusual metallic behavior is further corroborated by quantum‐mechanical first‐principles solid‐state calculations, providing compelling theoretical support for the experimentally observed transport characteristics. Collectively, these results represent a fundamental advance in metal–organic framework electronics and offer a promising platform for the rational design of next‐generation conductive metal–organic materials.

### 3.4 Photoconductivity

Upon light illumination, MOF materials generate a photocurrent through the photoexcitation of charge carriers from the highest occupied crystal orbital (HOCO) to the lowest unoccupied crystal orbital (LUCO) [46]. Figure 4a presents the photocurrent response curves of Cu‐MOF‐1 at 808 nm wavelength under different optical powers. Corresponding photocurrent responses measured at 325, 405, and 532 nm under different light intensities are shown in Figure S2 (ESI). Figure 4b depicts the photocurrent (*i*_p_) as a function of incident light intensity (*I*_L_) at different wavelengths. Notably, the photocurrent displays a linear dependence on light intensity, indicating a wide linear dynamic range (LDR) spanning 40–2000 W/m^2^, a critical performance metric for photodetector and image sensor applications.

**FIGURE 4:**
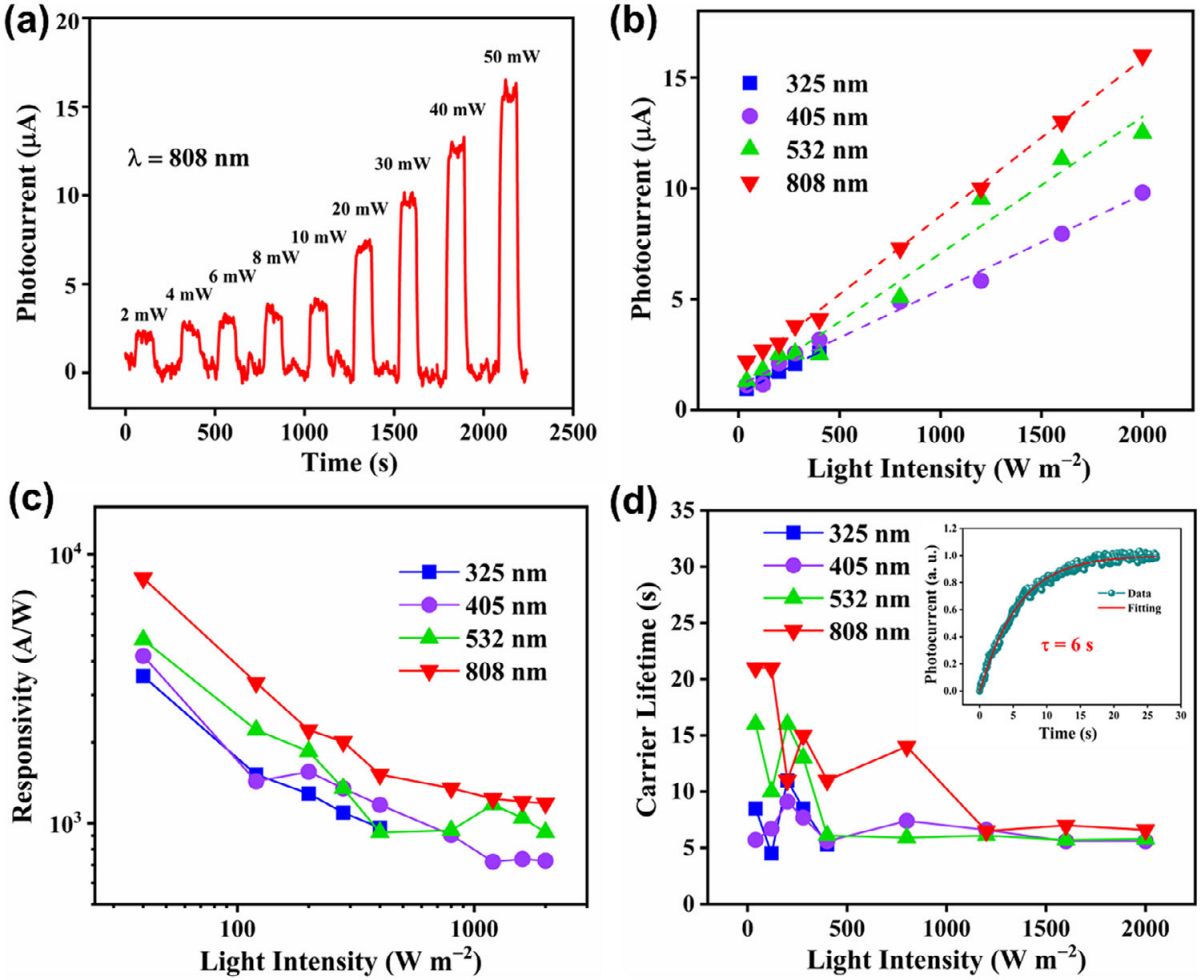
(a) Photoresponse curves of a Cu‐MOF single‐crystal device recorded at a bias voltage of 1 V under 808 nm illumination with increasing optical power. The background (dark) current was subtracted to clearly display the photocurrent response. Light‐intensity‐dependent plots of (b) photocurrent, (c) responsivity, and (d) carrier lifetime measured at excitation wavelengths of 325, 405, 532, and 808 nm. The dashed colored lines in panel (b) represent the linear fits to the corresponding experimental data. The inset in panel (d) shows the curve fitting for a typical photocurrent rising curve.

To evaluate the photodetector performance, the photocurrent responsivity (*R*) of the MOF device was determined. Responsivity is a key figure of merit that quantifies the efficiency of photocurrent generation per unit incident optical power and is defined as *R* = (*i*_p_/*P*), where *i*_p_ is the photocurrent and *P* denotes the incident optical power impinging on the effective device area [47].

The calculated responsivity (*R*) as a function of incident light intensity at different wavelengths is presented in Figure 4c. Notably, *R* increases with decreasing light intensity, reaching a maximum value of 8100 A W^−1^ at an incident power density of 40 W m^−2^ under 808 nm illumination. This exceptional responsivity significantly exceeds those reported for other organic semiconductor single crystals, including MBT (*R* ≈ 243 A W^−1^) [15], TPPB (*R* ≈ 88 A W^−1^) [16], and MDMO‐PPV (*R* ≈ 40 A W^−1^) [48], as well as inorganic In_x_Al_1‐x_N nanorods (*R* ≈ 1400 A W^−1^) [49] and representative two‐dimensional materials, such as MoS_2_ (*R* ≈ 30 A W^−1^) [50], NbSe_2_ (*R* ≈ 3.8 A W^−1^) [47], and NbS_2_ (*R* ≈ 0.6 A W^−1^) [51]. Because both responsivity and electrical conductivity are closely related to charge‐carrier mobility, the high responsivity is fully consistent with the high electrical conductivity of this Cu‐MOF. These results further imply a high charge drift velocity within the single crystal, underscoring the exceptional optoelectronic performance of this material.

Theoretically, responsivity is linearly dependent on photoconductive gain (*Γ*), i.e. *R* = *Γeη*/*E*, where *e* is the elementary charge, *η* is the quantum efficiency (assumed *η* = 1 to simplify the calculation), and *E* is the photon energy. The *Γ* value can be derived from the equation *Γ* = *ER*/*eη*. Consequently, the calculated Γ values of the Cu‐MOF‐1 sample reach as high as 1500–13500 at a wavelength of 808 nm over the intensity range of 40–2000 W m^−2^. Photoconductive gain, a critical figure of merit for evaluating photocarrier collection efficiency in photoconductors, is defined as the ratio of the carrier lifetime (*τ*) to the transit time between electrodes (*τ_t_*), expressed as *Γ* = *τ* /*τ*_t_ = *τ*µV/*l* ^2^, where *µ* is the carrier mobility [52, 53]. According to the equation, *Γ* is highly dependent on the intrinsic material properties, such as *τ* and *μ*. Time‐resolved photocurrent measurements were performed to elucidate the carrier lifetime and to probe the physical origin of the high responsivity and gain. According to the continuity equation, the photocurrent (*i_p_*) follows the relation *i_p_* ∝[1−exp(−*t*/*τ*)], where *t* denotes the rise time of the photoresponse and τ corresponds to the carrier lifetime. The *τ* values were extracted by exponential fitting of the time‐resolved photocurrent traces (inset of Figure 4d). Figure 4d summarizes the extracted carrier lifetimes as a function of incident light intensity at different excitation wavelengths. For 808 nm light, the carrier lifetimes are remarkably long, spanning 6–21 s, indicative of highly suppressed carrier recombination. Notably, these lifetimes are orders of magnitude longer than those typically observed in conventional inorganic semiconductors, such as Si and GaAs, where the carrier lifetimes generally fall within the microsecond to nanosecond scale. The extended carrier lifetime thus accounts for the enhanced responsivity observed in the Cu‐MOF device. The extraordinarily long carrier lifetime is likely attributed to the presence of carrier traps in the MOF crystals. For instance, the lifetime of unpaired electrons could be substantially prolonged when the excess holes are captured by the minority traps. Further studies are required to elucidate the underlying mechanism.

### 3.5 Theoretical First‐Principles Calculations

To gain atomic‐level insight into the electronic properties of the Cu‐MOF single crystal, we examined its fundamental characteristics, such as band structure, bandgap, and density of states (DOS) using first‐principles calculations based on density functional theory (DFT) as implemented in the VASP code [34, 35]. The spin‐polarized band structures (Figure 5), reveal markedly different electronic behaviors for the spin‐up (majority spins) and spin‐down (minority spins) channels, providing clear evidence of half‐metallicity (Figure 5a). In the spin‐up (↑) channel, multiple bands cross the Fermi‐level, confirming metallic behavior (Figure 5b). In contrast, the spin‐down (↓) channel exhibits semiconducting behavior with a narrow bandgap of 0.63 eV (Figure 5c). Notably, the valence band dispersion in the semiconducting channel is exceptionally steep (Figure S3a, ESI), with a sharp decline along the Γ–E (or Γ–Y) direction of the Brillouin zone. The energy difference between Γ and E reaches 0.81 eV, approximately twice that reported in our previous study and considered remarkably high for MOF‐based semiconductors [12]. This steep dispersion leads to yielding a reduced effective mass, which is expected to enhance charge‐carrier mobility and, consequently, electrical conductivity. Collectively, these results demonstrate that the Cu‐MOF single crystal simultaneously exhibits metallic and semiconducting electronic states, depending on the spin channel. It is worth emphasizing that half‐metallicity is exceedingly rare in MOF materials, and its realization in this system offers significant opportunities for advanced applications in optoelectronics, spintronics, and quantum technologies [24, 25].

**FIGURE 5:**
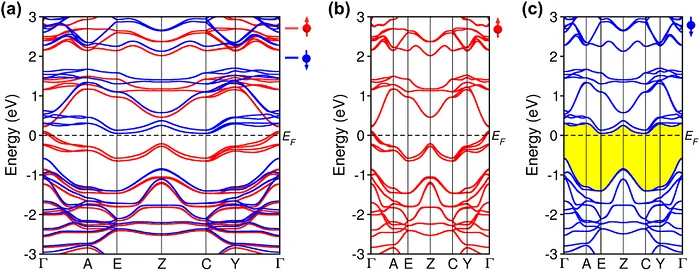
(a) Spin‐polarized band structures of Cu‐MOF crystal: (b) the majority and (c) minority spin channels. Red (blue) lines represent the spin‐up (down) bands. The dashed line illustrates the Fermi‐level, *E*_F_.

To elucidate the atomic‐level origin of the coexistence of metallic and semiconducting behavior, we analyzed the spin‐resolved density of states (DOS) of the Cu‐MOF single crystal (Figure 6). The total DOS is strongly spin‐polarized, exhibiting pronounced asymmetrical features between the majority‐spin (spin‐up) and minority‐spin (spin‐down) channels (Figure 6a). The metallic character in the spin‐up channel arises from contributions of both the organic linkers and Cu metal nodes (Figure 6b), whereas the semiconducting behavior in the spin‐down channel is primarily associated with the orbital hybridizations of Cu and S atoms (Figure 6c). Furthermore, band‐decomposed partial charge‐density analyses reveal that (–Cu–S–)_n_ chains provides a dense spatial distribution of charge carrier (Figure S3b, ESI). These computational results demonstrate that the (–Cu–S–)_n_ chains play a significant role in charge transport, consistent with the experimentally observed high electrical conductivity. Note that the half‐metallic Cu‐MOF material, characterized by spin‐polarized electronic structures with one spin channel metallic and the other semiconducting, is of exceptional interest in spintronics and quantum technologies. This study not only introduces a new concept based on quantum spin‐polarized electronic structure but also represents a novel strategy for developing next‐generation conductive materials.

**FIGURE 6:**
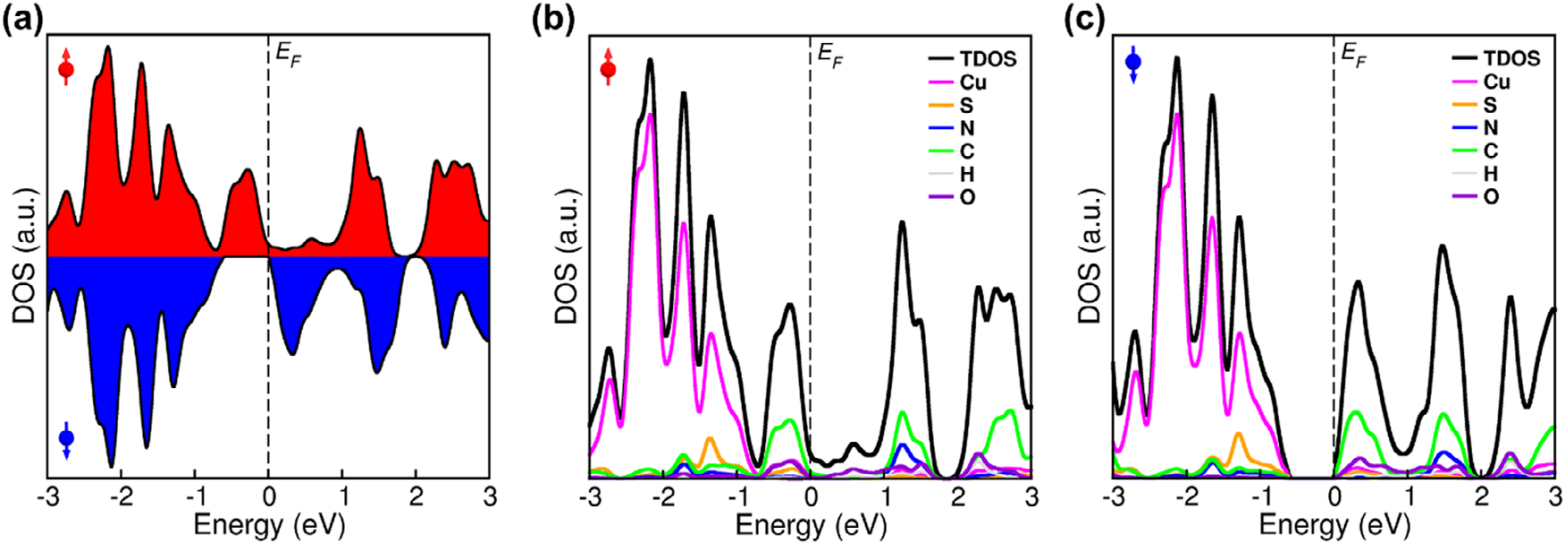
(a) Spin‐polarized DOS of the Cu‐MOF crystal along the (b) spin‐up and (c) spin‐down channel. Red (blue) arrow indicates the spin‐up (down) channel. The dashed line illustrates the Fermi‐level, *E*_F_.

## 4 Conclusions

The Cu‐MOF single crystal exhibits a high electrical conductivity of 120 S cm^−1^ and displays stable and reproducible photoconductive behavior under infrared illumination, achieving an optimal responsivity of 8100 A W^−1^ at a light intensity of 40 W m^−^^2^. Temperature‐dependent conductivity measurements unambiguously confirm the metallic transport behavior of the Cu‐MOF crystals, in excellent agreement with the corresponding theoretical calculations. Our results reveal that spin polarization an electronic degree of freedom largely overlooked in MOFs plays a decisive role in enhancing charge transport. Notably, this mechanism enables the first demonstration of half‐metallicity in a MOF with such high conductivity, thereby integrating fundamental quantum spin effects into the design principles of conductive frameworks. Collectively, these findings redefine the understanding of MOF conductivity by establishing spin as a key quantum descriptor of electronic transport, while simultaneously opening new opportunities for advanced applications in electronics, spintronics, and quantum technologies. Moreover, this work introduces a new materials design paradigm that synergistically combines scalable synthesis with intrinsic quantum functionality.

## Author Contributions

**Saqib Kamal**: investigation, Writing – original draft, methodology, validation, visualization. **Kuang‐Hsin Hsu**: data curation, validation, formal analysis. **Arif I. Inamdar**: investigation, validation, methodology, data curation. **Hemanth Kumar Bangolla**: validation, data curation, formal analysis, investigation. **Chi‐Jia Lin**: validation, data curation. **Batsaikhan Erdembayalag**: software, visualization, data curation. **Tzuoo‐Tsair Luo**: validation, methodology. **Ming‐Hsi Chiang**: validation, supervision. **Yen‐Hsiang Liu**: data curation, validation. **Ruei‐San Chen**: resources, methodology, validation, investigation, writing – review and editing. **Sainbileg Batjargal**: conceptualization, investigation, funding acquisition, writing – original draft, methodology, validation, visualization, writing – review and editing, software, supervision, formal analysis, resources. **Michitoshi Hayashi**: software, methodology, resources. **Chen‐Hsiung Hung**: funding acquisition, supervision, validation. **Kuang‐Lieh Lu**: conceptualization, funding acquisition, supervision, writing – review and editing, project administration, resources.

## Conflicts of Interest

The authors declare no conflicts of interest.

## Supporting information




**Supporting File**: advs78726‐sup‐0001‐SuppMat.docx.

## Data Availability

The data that support the findings of this study are available from the corresponding author upon reasonable request.
