# Supplementary material for: Spin‐Polarized Metallic Metal−Organic Framework With High Conductivity and Half‐Metallic Behavior
Source: Adv Sci (Weinh). 2026 Nov 9:e78726. Online ahead of print. doi: 10.1002/advs.78726 (PMC13748892; doi:10.1002/advs.78726)
Supplement: Supplementary file 1 [file ADVS-9999-e78726-s001.docx]

**Supplementary Information**

**Spin-Polarized Metallic Metal−Organic Framework with** **High Conductivity and Half-Metallic Behavior**

Saqib Kamal,^1,2,3^ Kuang-Hsin Hsu,^4^ Arif I. Inamdar,^1^ Hemanth Kumar Bangolla,^4^ Chi-Jia Lin,^4^ Batsaikhan Erdembayalag,^5,6^ Tzuoo-Tsair Luo,^1^ Ming-Hsi Chiang,^1^ Yen-Hsiang Liu,^2^ Ruei-San Chen,*^,4^ Sainbileg Batjargal,*^,5,6^ Michitoshi Hayashi,*^5,6,7^ Chen-Hsiung Hung*^,1^ and Kuang-Lieh Lu*^,1,2^

^1^Institute of Chemistry, Academia Sinica, Taipei 115, Taiwan

^2^Department of Chemistry, Fu Jen Catholic University, New Taipei City 242, Taiwan

^3^Department of Chemistry, Emerson University Multan, Multan 60000, Pakistan

^4^Graduate Institute of Applied Science and Technology, National Taiwan University of Science and Technology, Taipei 106, Taiwan

^5^Center for Condensed Matter Sciences, National Taiwan University, Taipei 106, Taiwan

^6^Center of Atomic Initiative for New Materials, National Taiwan University, Taipei 106, Taiwan

^7^National Center for Theoretical Sciences, Taipei 106, Taiwan

Corresponding author email: [batjargal@ntu.edu.tw](mailto:batjargal@ntu.edu.tw)

**Figure S1.** Bonding environment of the Cu(I) ion, showing the long-range interchain Cu···Cu interactions (3.064 Å) and the coordination mode of the 6Hmna ligand within the crystal packing of Cu-MOF **1**. Atom color code: Cu = pink, S = yellow, N = blue, O = green.

**Figure S2.** Photoresponse curves of a Cu-MOF single crystal recorded at a bias of 1 V, escalating optical power, and at wavelengths of (a) 325, (b) 405, and (c) 532 nm. The background current was subtracted from the measured photoresponse curves to present clear photocurrent values.

**Figure S3.** (a) Band structure of the Cu-MOF crystal in the spin-down channel, along with a zoomed-in view of its valence band, exhibits a steep dispersion. (b) Valence band edge feature: the band-decomposed partial charge density (green colored isosurfaces) shows VBM originates from the electronic states of Cu and S in the (–Cu–S–)_n_ chain of the crystal (Atom color code: Cu = brown, S = yellow, N = blue, O = red, C = grey).

**Table S1. Crystal data and structure refinement for Cu-MOF 1**

Identification code	ic19999

Empirical formula	C6 H4 Cu N O2 S

Formula weight	217.70

Temperature	200(2) K

Wavelength	0.71073 Å

Crystal system	Monoclinic

Space group	*P*2**_1_**/*c*

Unit cell dimensions	*a* = 3.8928(2) Å	α = 90°

*b* = 5.9059(4) Å	β = 92.8568(15)°

*c* = 27.8772(14) Å	γ = 90°

Volume	640.11(6) Å^3^

*Z*	4

Density (calculated)	2.259 Mg/m^3^

Absorption coefficient	3.667 mm^-1^

*F*(000)	432

Crystal size	0.214 x 0.120 x 0.015 mm^3^

Theta range for data collection	2.926 to 27.497°

Index ranges	-5<=h<=5, -7<=k<=7, -30<=l<=36

Reflections collected	4198

Independent reflections	1463 [R(int) = 0.0324]

Completeness to theta = 25.242°	99.8 %

Absorption correction	Semi-empirical from equivalents

Max. and min. transmission	0.7456 and 0.5324

Refinement method	Full-matrix least-squares on *F*^2^

Data / restraints / parameters	1463 / 12 / 109

Goodness-of-fit on *F*^2^	1.261

Final R indices [*I* > 2σ(*I*)]	R1 = 0.0496, wR2 = 0.0961

R indices (all data)	R1 = 0.0527, wR2 = 0.0972

Extinction coefficient	n/a

Largest diff. peak and hole	0.830 and -1.307 e.Å^-3^

**Table S2. Comparison of conductivity and activation energy of our Cu-MOF crystal with other MOF and metal–organic single crystals.**

| **Compound** | **Conductivity (S cm^−1^)** | **Contact Configuration** | **Activation energy (meV)** | **Reference** |
| --- | --- | --- | --- | --- |
| [Cu(6Hmna)]_n_ | 120 | Two probe | 0 | **This work** |
| Cd_2_(TTFTB) | 2.5 × 10^−4^ | Two probe | 293 | 1 |
| Fe_2_(BDT)_3_ | 1.2 | Two probe | 160 | 2 |
| [{Re-(CO)_3_}_6_(bho)(phpy)_6_]_n_ | 1.5 | Two probe | 1.6 | 3 |
| [Zn(TPPB)(Cl_2_)] H_2_O | 1.9 | Two probe | 18 | 4 |
| {[Cu_2_(6-Hmna) (6-mn)]·NH_4_}_n_ | 10.9 | Four probe | 6 | 5 |
| RePy-MONT | 2.3 | Two probe | 8.4 | 6 |

**Supplementary References:**

1. L. Sun, S. S. Park, D. Sheberla, M. Dincǎ, Measuring and Reporting Electrical Conductivity in Metal–Organic Frameworks: Cd_2_(TTFTB) as a Case Study. *J. Am. Chem. Soc.* *138*, (2016): 14772–14782.
2. L. S. Xie, L. Sun, R. Wan, S. S. Park, J. A. DeGayner, C. H. Hendon, M. Dincă, Tunable Mixed-Valence Doping toward Record Electrical Conductivity in a Three-Dimensional Metal–Organic Framework. *J. Am. Chem. Soc.* *140*, (2018): 7411–7414.

3.	A. I. Inamdar, B. Sainbileg, C.-J. Lin, M. Usman, S. Kamal, K.-R. Chiou, A. Pathak, T.-T. Luo, K. S. Bayikadi, R. Sankar, J.-W. Chen, T.-W. Tseng, R.-S. Chen**,** M. Hayashi**,** M.-H. Chiang, K.-L. Lu, Regimented Charge Transport Phenomena in Semiconductive Self-Assembled Rhenium Nanotubes. *ACS Appl. Mater. Interfaces* *14*, (2022): 12423–12433.

4.	C. M. Ngue, K. F. Ho, B. Sainbileg, [E. Batsaikhan](https://pubs.rsc.org/en/results?searchtext=Author%3AErdembayalag%20Batsaikhan), [M. Hayashi](https://pubs.rsc.org/en/results?searchtext=Author%3AMichitoshi%20Hayashi), [K. Y. Lee](https://pubs.rsc.org/en/results?searchtext=Author%3AKuei%20Yi%20Lee), [R. S. Chen](https://pubs.rsc.org/en/results?searchtext=Author%3ARuei%20San%20Chen), [M. K. Leung](https://pubs.rsc.org/en/results?searchtext=Author%3AMan%20Kit%20Leung), Conductivity and photoconductivity in a two-dimensional zinc bis(triarylamine) coordination polymer. *Chem. Sci.* *14*, (2023): 1320–1328.

5.	[A. Pathak](https://www.nature.com/articles/s41467-019-09682-0#auth-Abhishek-Pathak-Aff1-Aff2-Aff3), [J.-W. Shen](https://www.nature.com/articles/s41467-019-09682-0#auth-Jing_Wen-Shen-Aff4), [M. Usman](https://www.nature.com/articles/s41467-019-09682-0#auth-Muhammad-Usman-Aff1), [L.-F. Wei](https://www.nature.com/articles/s41467-019-09682-0#auth-Ling_Fang-Wei-Aff1), [S. Mendiratta](https://www.nature.com/articles/s41467-019-09682-0#auth-Shruti-Mendiratta-Aff1), [Y.-S. Chang](https://www.nature.com/articles/s41467-019-09682-0#auth-Yu_Shin-Chang-Aff5), [B. Sainbileg](https://www.nature.com/articles/s41467-019-09682-0#auth-Batjargal-Sainbileg-Aff6-Aff8), [C.-M. Ngue](https://www.nature.com/articles/s41467-019-09682-0#auth-Chin_May-Ngue-Aff1), [R.-S. Chen](https://www.nature.com/articles/s41467-019-09682-0#auth-Ruei_San-Chen-Aff5), [M. Hayashi](https://www.nature.com/articles/s41467-019-09682-0#auth-Michitoshi-Hayashi-Aff6-Aff8), [T.-T. Luo](https://www.nature.com/articles/s41467-019-09682-0#auth-Tzuoo_Tsair-Luo-Aff1), [F.-R. Chen](https://www.nature.com/articles/s41467-019-09682-0#auth-Fu_Rong-Chen-Aff2), [K.-H. Chen](https://www.nature.com/articles/s41467-019-09682-0#auth-Kuei_Hsien-Chen-Aff6-Aff7), [T.-W. Tseng](https://www.nature.com/articles/s41467-019-09682-0#auth-Tien_Wen-Tseng-Aff4), [L.-C. Chen](https://www.nature.com/articles/s41467-019-09682-0#auth-Li_Chyong-Chen-Aff6-Aff8), [K.-L. Lu](https://www.nature.com/articles/s41467-019-09682-0#auth-Kuang_Lieh-Lu-Aff1), Integration of a (–Cu–S–)*_n_* Plane in a Metal–Organic Framework Affords High Electrical Conductivity. *Nat. Commun.* *10*, (2019): 1721.

6.	A. I. Inamdar, H. K. Bangolla, K.-F. Ho, B. Sainbileg, E. Batsaikhan, R.-S. Chen, M. Hayashi, M.-H. Chiang, K.-L. Lu, Band Gap Engineering, Dark Conductivity, and Photoconductive Properties of Semiconductive Self-Assembled Rhenium Nanotube by Structural Topology Modification. *ACS Appl. Electron. Mater.* ***7***, (2025): 533–541.
